# The Inhibitory Effect of Selected D_2_ Dopaminergic Receptor Agonists on VEGF-Dependent Neovascularization in Zebrafish Larvae: Potential New Therapy in Ophthalmic Diseases

**DOI:** 10.3390/cells11071202

**Published:** 2022-04-02

**Authors:** Natalia Kasica, Anna Święch, Katarzyna Saładziak, Jerzy Mackiewicz, Maciej Osęka

**Affiliations:** 1 Department of Animal Anatomy, Faculty of Veterinary Medicine, University of Warmia and Mazury in Olsztyn, Oczapowskiego 13 Street, Box 105J, 10-719 Olsztyn, Poland; 2 Department of Retina and Vitreus Surgery, Medical University in Lublin, Chmielna 1 Street, 20-079 Lublin, Poland; anna.zub@umlub.pl (A.Ś.); kadanieluk@gmail.com (K.S.); jerzy.mackiewicz@umlub.pl (J.M.); 3 Oftalabs Sp. z o.o., Wrocławska 130, 58-306 Wałbrzych, Poland; maciej_oseka@oftalabs.pl

**Keywords:** zebrafish, angiogenesis, D_2_ dopaminergic receptor agonist, bromocriptine, cabergoline, pergolide, hyaloid-retinal vessels (HRVs), intersegmental vessels (ISVs), vascular endothelial growth factor

## Abstract

Pathological angiogenesis is correlated with many ophthalmic diseases. The most common are exudative age-related macular degeneration and proliferative diabetic retinopathy. The current treatment for these diseases is based on regularly administered anti-VEGF antibodies injections. In the study, we investigated selected D_2_ dopaminergic receptor agonists, namely bromocriptine, cabergoline and pergolide, on hypoxia-induced neovascularization. We used the zebrafish laboratory model, specifically three-day post fertilization (dpf) Tg(*fli*-*1*: EGFP) zebrafish larvae. To induce abnormal angiogenesis of hyaloid-retinal vessels (HRVs) and intersegmental vessels (ISVs), the larvae were treated with cobalt chloride (II) (CoCl_2_) (a hypoxia-inducing agent) from 24 h post fertilization. The inhibitory role of D_2_ dopaminergic receptor agonists was investigated using confocal microscopy and qPCR. Additionally, the results were compared to those obtained in the group treated with CoCl_2_ followed by bevacizumab, the well-known antiangiogenic agent. Confocal microscopy analyses revealed severe deformation of vessels in the CoCl_2_ treated group, while co-incubation with bromocriptine, cabergoline, pergolide and bevacizumab, respectively, significantly inhibited abnormalities of angiogenesis. The qPCR analyses supported the protective role of the chosen dopaminergic agonists by demonstrating their influence on CoCl_2_-derived upregulation of *vegfaa* expression. The present results suggest that the D_2_ receptor agonists can be considered as a new direction in research for antiangiogenic therapy.

## 1. Introduction

Vascular endothelial growth factor (VEGF) is a heparin-binding, dimeric protein responsible for the development and maintenance of the vascular and the lymphatic systems. Its production and release can be stimulated by acute or chronic hypoxia. VEGF triggers endothelial cells to extracellular matrix degradation, proliferation and migration [1]. It also increases vascular permeability and acts as an endothelial cell survival factor inducing new vessel formation [2]. Despite the advantages provided by the physiological level of VEGF, its overexpression can lead to selected retinal diseases, which are responsible for serious vision impairment or blindness. Stimulated by VEGF, new formation of choroidal vessels and their penetration through retinal pigment epithelium (RPE) into the retina is characteristic of the exudative form of age-related macular degeneration (eAMD). VEGF is also responsible for the increased permeability and neovascularization of retinal blood vessels in the proliferative form of diabetic retinopathy (PDR) and diabetic macular edema (DME). High production and release of VEGF due to retinal artery or vein occlusion and tissue hypoxia are a cause of retinal edema and proliferation of retinal vessels in later stages of those diseases [3]. It seems that VEGF is also involved in etiopathogenesis of retinopathy of prematurity (ROP) [4].

Currently, intravitreal injections of monoclonal antibodies against VEGF and/or its receptor (anti-VEGFs) are used in the treatment of the ophthalmic diseases. Due to the complicated procedure of anti-VEGFs intraocular application, various efficacies of the treatment in long-term perspective and risk of side effects, there is still a need to develop new drugs with anti-VEGF effects as alternative or supportive treatments of the diseases mentioned above.

Epidemiological studies have shown an increased risk of eAMD in people with Parkinson’s disease and vice versa [5]. It was also noticed that the risk of eAMD is significantly reduced in patients with Parkinson’s disease treated with dopamine precursors [6]. Those observations suggest the potential effect of dopamine or dopaminergic receptor agonists in the inhibition of eAMD onset and progression.

Based on the available data, we considered three D_2_ dopaminergic receptor agonists—cabergoline, bromocriptine and pergolide—to be promising candidates for eAMD and PDR treatment. Cabergoline and bromocriptine were already screened for their anti-VEGF effect in the course of other diseases, such as ovarian hyperstimulation syndrome (OHSS). It was revealed that both substances can suppress the rise of VEGF level, decrease capillary permeability of vessels and stop a shift of intravascular fluid to the third space in the abdominal cavity [7]. Pergolide is another dopaminergic agonist, which in the past was used as a treatment in horse hyperprolactinemia and Parkinson’s disease [8,9]. Pergolide has not yet been tested for its antiangiogenic effect; however, considering its similar biological structure to the above-mentioned dopaminergic agonists, it presumably shares similar properties.

The aim of this study was to test the influence of chosen D_2_ dopaminergic receptor agonists on hypoxia-derived vasculature disruption. For this purpose, we used a zebrafish laboratory model. Zebrafish is a very popular model in new drug testing. It is relatively cheap and easy to breed. When kept under the optimal conditions, zebrafish can produce hundreds of offspring weekly. Embryo and larva stages have a transparent body that is easily accessible for observation. Moreover, it serves as an excellent model for angiogenesis research [10]. Zebrafish angiogenesis starts with differentiation of hemangioblasts from mesoderm, followed by differentiation of angioblasts and endothelial cells [11,12]. By 24 h post-fertilization (hpf), there is a simple circulatory system, consisting of axial vein, dorsal aorta and ducts of Cuvier [13]. Intersegmental vessels (ISVs) of the trunk and the tail are formed by 48 hpf and most other vessels by 72 hpf. This early development of the circulatory system in transparent embryo and larva gives an opportunity for direct in vivo observing and imaging of single vessels and related organs.

Considering the above-mentioned issues, the present study was designed to establish the role of bromocriptine, cabergoline and pergolide towards cobalt chloride (II) (CoCl_2_)-derived improper vessel development in zebrafish larvae. The goal of the study was to find morphological differences in newly formed hyaloid-retinal (HRVs) and intersegmental vessels (ISVs) and to investigate whether chosen dopaminergic agonists regulate the gene expression of VEGF and VEGF-R2. The obtained data could provide a new direction in the treatment of angiogenic abnormalities in eAMD and PDR.

## 2. Materials and Methods

### 2.1. Zebrafish Embryos Care and Maintenance

The study involved 3 day post-fertilization (dpf) zebrafish larvae of the transgenic line Tg(Fli-1:EGFP), which carries Fli-1 proto-oncogene/ETS transcription factor promoter driving the expression of green fluorescent protein (GFP) in the blood vessels. Adult parental fish were maintained at 28 °C with a 14 h light:10 h dark photoperiod and fed three times daily ad libitum with dry food and *Artemia* sp. naupli. Embryos were maintained in an embryo solution (E3 medium) (5 mM NaCl, 0.17 mM KCl, 0.33 mM CaCl_2_, 0.33 mM MgSO_4_) or test solutions and were kept in an incubator at 28.5 °C and 14 h light:10 h dark cycle without feeding.

### 2.2. Drug Administration

To establish the influence of D_2_ dopaminergic receptor agonists (bromocriptine, cabergoline and pergolide (Merck, Darmstadt, Germany)) on cobalt (II) chloride hexahydrate (CoCl_2_ 6H_2_O; Warchem, Warsaw, Poland)-induced neovascularization and vascular defects, shortly after fertilization embryos were randomly assigned to 6 groups: (1) a control group including embryos which were incubated in embryo solution (E3 medium); (2) a group exposed to 5 mM CoCl_2_; (3) a group exposed to a mixture of 5 mM CoCl_2_ and 2.5 µM/L bromocriptine; (4) a group exposed to a mixture of mM CoCl_2_ and 2.5 µM/L cabergoline; (5) a group exposed to a mixture of 5 mM CoCl_2_ and 2.5 µM/L pergolide; and (6) a group exposed to a mixture of 5 mM CoCl_2_ and 2.5 µM/L bevacizumab. The incubation with dopaminergic agonists and bevacizumab (Roche, Basel, Switzerland) started at 10 hpf. In our study we used bevacizumab as a control solution with already established protective influence towards abnormal blood vessel formation. At 24 hpf, the embryos were co-treated with 5 mM CoCl_2_. The treatment lasted until 3 dpf. Every 24 h, the solutions were replaced with new ones.

### 2.3. Phenotype-Based Evaluation of Trunk and Retinal Vascularization

The visualization and images of trunk vessels were achieved using an LSM 700 confocal laser scanning microscope. To obtain desirable quality of images, ×20 objectives and the z-stack tool were applied. Stacks of images were composed into one to obtain maximum intensity projection images with ZEN 2009 software (Zeiss, Oberkochen, Germany). The analyses were restricted to ISVs. The evaluation was accomplished by quantification of each abnormal vessel (not fully formed or branched). In the case of HRVs, a Stereo discovery V8 (Zeiss, Germany) equipped with a DLT-Cam PRO 6.3 MP (Delta Optical, Warsaw, Poland) camera was used. Adobe Photoshop 7.0 software (Adobe, San Jose, CA, USA) was used to adjust the level, brightness and contrast of a series of images to optimize the visual representation. In both cases the GFP driven by Fli-1 proto-oncogene/ETS transcription factor promoter in the Tg(Fli1a:EGFP) line was excited by a 488 nm laser. To take images, the 3 dpf larvae were anesthetized with 0.02% MS-222 solution and mounted on glasses in a drop of 3% methylcellulose. Images of HRVs were analyzed by SketchandCalc software (iCals Inc. Whitinsville, MA, USA). The total area of HRVs was marked in each image and calculated by the software. To eliminate the risk of errors caused by various distances of the camera from the zebrafish eyeball, the results were presented as a kappa (ĸ) coefficient. The kappa (ĸ) coefficient was calculated as a ratio of HRVs area to eyeball area.

### 2.4. RNA Extraction, Reverse Transcription and qPCR Analysis

Gene expression analysis was performed in 3 dpf zebrafish larvae. After the 3 days of incubation in test solutions (described above) zebrafish were pooled (n = 30), frozen and stored in −80 °C. Larvae were homogenized by a TissueLyser II (Qiagen, Dusseldorf, Germany). Total RNA was extracted using a Total RNA Mini isolation kit (AA Biotechnology, Gdynia, Poland). All steps of isolation were assessed according to the respective manufacturer’s protocols. The cDNA samples were synthesized from respective high quality matrix samples with equal RNA concentration for each sample using a Maxima First Strand cDNA Synthesis Kit for RT-qPCR (Thermo Scientific, Waltham, MA, USA). All steps of reverse transcription were assessed according to the manufacturers’ protocols. qPCR was performed using SYBR Green (SYBR Select Master Mix, Applied Biosystems, Foster City, CA, USA) on a 7500 Fast Real-Time PCR System instrument (Applied Biosystems, Foster City, CA, USA) under previously described conditions [14]. Oligonucleotide primers were selected to detect: *vascular endothelial growth factor Aa* (*vegfaa*), *vascular endothelial growth factor receptor 1* (*vegfr1*), *vascular endothelial growth factor receptor 2* (*vegfr2*), *placental growth factor a* (*pgfa*), *placental growth factor b* (*pgfb*), *fibroblast growth factor 2* (*fgf2*), *transforming growth factor-beta 1* (*tgfb1*), *insulin growth factor 2a* (*igf2a*) and *insulin growth factor 2b* (*igf2b*). The details are listed in Table 1. Initial validation of reference genes revealed that for the purpose of the study, *elongation factor 1 alfa* *(ef1-α)* showed the most efficient and equal expression among the samples. The values of the expression of the studied genes were calculated in each group as a relative expression to *ef1-α*. Each sample was analyzed in triplicate in three separate experiments.

### 2.5. Statistical Analysis

The statistical analysis was performed using GraphPad Prism, version 5.0 (GraphPad Software Inc., San Diego, CA, USA). Data with assumed Gaussian distribution were analyzed using a one-way ANOVA test with Tukey multiple comparisons tests as a post hoc test or Student’s t test. Data analyses not assuming Gaussian distribution were based on a Kruskal–Wallis test with Dunn’s multiple comparisons test as a post hoc test. The error bars represent means ± SEM. The significance level was set at *α* = 0.05 (95% confidence intervals).

## 3. Results

### 3.1. Inhibitory Effects of Selected D_2_ Dopaminergic Receptor Agonists towards CoCl_2_-Derived Increase of Hyaloid-Retinal Vessels

**Figure 1:**
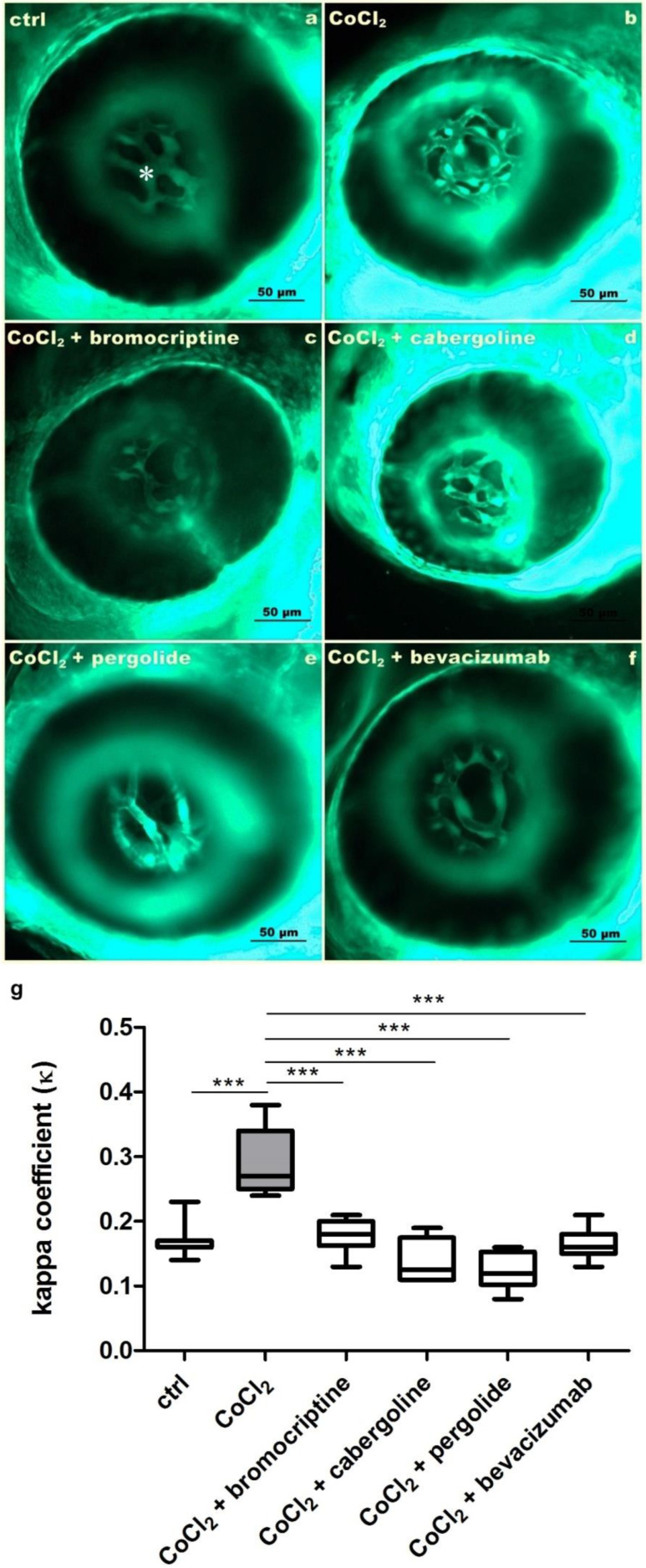
A set of microphotographs and a graph documenting inhibition of 5 mM CoCl_2_-derived increased angiogenesis of hyaloid-retinal vessels (HRVs) (asterisk) resulting from co-treatment with dopaminergic agonists in 3 dpf Tg(*Fli*-*1*:EGFP) zebrafish larvae. (**a**) The control untreated larva; HRVs are marked with *. (**b**) 5 mM CoCl_2_ exposure resulted in increased HRVs branching. (**c**–**g**) Co-treatment with 2.5 µM/L bromocriptine, 2.5 µM/L cabergoline, 2.5 µM/L pergolide and 2.5 µM/L bevacizumab, respectively, resulted in significant inhibition of HRVs abnormal branching. (**g**) The graph presenting the kappa coefficient (ĸ) values (ratio of HRVs area to eyeball area) in investigated groups. (one-way ANOVA, GraphPad Prism 5, *** *p* < 0.001).

Exposure to 5 mM CoCl_2_ resulted in multiplication of HRVs branching leading to increased kappa coefficient (ĸ) (Figure 1b,g). This effect was significant in comparison with the control conditions (Figure 1a,g) (*p* < 0.001). The co-treatment with bromocriptine (Figure 1c), cabergoline (Figure 1d) or pergolide (Figure 1e) blocked hypoxia-induced neovascularization, and this effect was clearly reflected by decreased kappa coefficient (ĸ) values (Figure 1g) (*p* < 0.001). The observed effects were also comparable with those obtained in the group co-treated with the anti-VEGF monoclonal antibody bevacizumab (Figure 1f,g).

### 3.2. Ameliorative Effects of Selected D_2_ Dopaminergic Receptor Agonists towards CoCl_2_-Derived Trunk Blood Vessels Disruption

**Figure 2:**
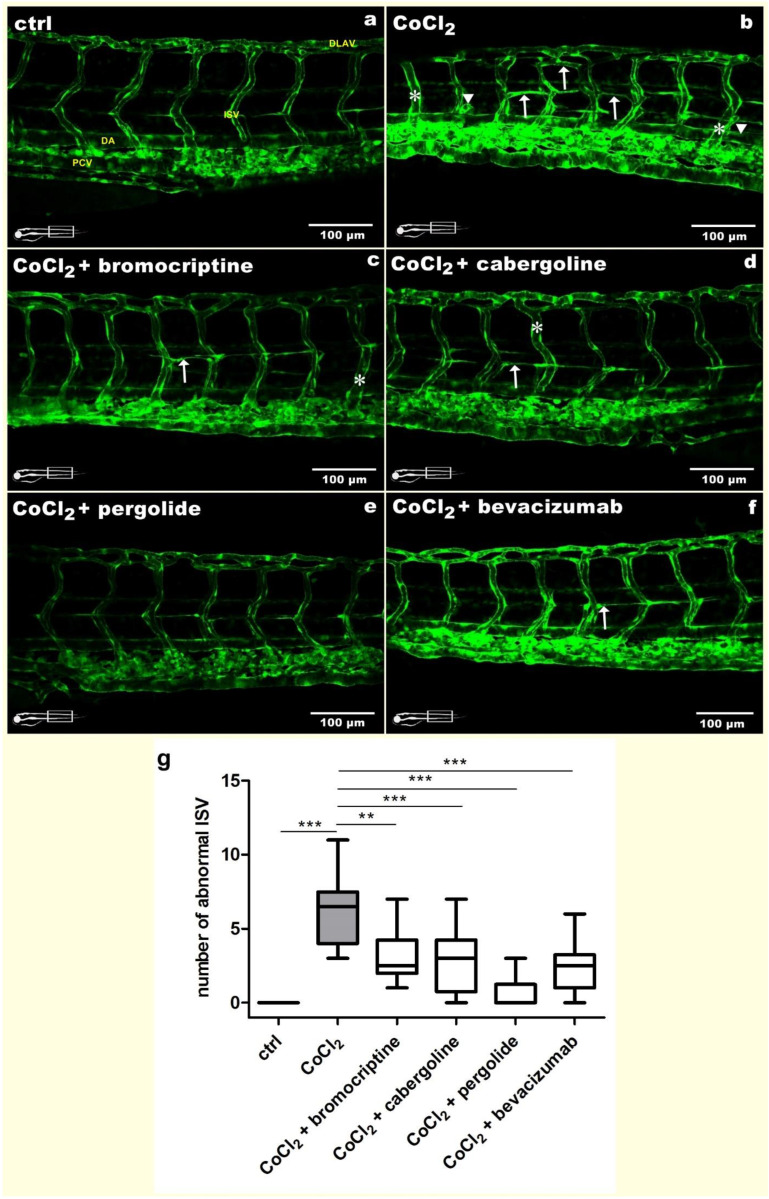
A set of microphotographs and a graph documenting inhibition of 5 mM CoCl_2_-derived abnormal angiogenesis of intersegmental vessels (ISVs) resulting from co-treatment with dopaminergic agonists in 3 dpf Tg(*Fli*-*1*:EGFP) zebrafish larvae. (**a**) The control untreated larva presented normal morphology and distribution of ISVs; (**b**) 5 mM CoCl_2_ exposure promoted increased protrusive/regressive activity (arrowhead), branching (arrow) and swelling (asterisk). (**c**–**f**) Co-treatment with 2.5 µM/L bromocriptine, 2.5 µM/L cabergoline, 2.5 µM/L pergolide and 2.5 µM/L bevacizumab, respectively, resulted in significant inhibition of improper ISVs development. (**g**) The graph presenting the number of all abnormalities found in investigated groups (one-way ANOVA, GraphPad Prism 5, *** *p* < 0.001; ** *p* < 0.01). PCV—posterior cardinal vein; DA—dorsal aorta; ISV—intersegmental vessel; DLAV—dorsal longitudinal anastomotic vessel.

To support the protective effects of dopaminergic agonists observed in the retina, the phenotype of trunk blood vessels was assessed. The control blood vessels showed normal and regular morphology (Figure 2a). Exposure to 5 mM CoCl_2_ did not alter large vessels such as the posterior cardinal vein (PCV) or dorsal aorta (DA); however, it evoked distinct changes in intersegmental vessels (ISVs) (Figure 2b). Hypoxic conditions promoted protrusive/regressive activity, expressed as short, not fully developed sprouts (arrowheads). Moreover, in the 5 mM CoCl_2_ exposed group, the ISVs were branched and created commeasures (arrow). The rarest but indisputable observation concerns swelling of individual vessels (asterisk). The average number of abnormal ISV in this group was 6.4 per larvae (Figure 2g). We also observed disruptions in the dorsal longitudinal anastomotic vessel (DLAV), which did not form regular loops (Figure 2b). Co-treatment with the chosen dopamine D_2_ receptor agonists resulted in reducing improper vascularization. The best effects were obtained in the 2.5 µM/L pergolide co-treated group, where we reported altered vessels with an average number of 0.7 per larvae (*p* < 0.001) (Figure 2e,g). The 2.5 µM/L bromocriptine (Figure 2c), 2.5 µM/L cabergoline (Figure 1d) and 2.5 µM/L bevacizumab (Figure 2f) treatments resulted in modest average amounts of abnormalities, equal to 3.2 (*p* < 0.01), 2.9 (*p* < 0.001) and 2.5 (*p* < 0.001) per larvae, respectively (Figure 2g).

### 3.3. Effects of CoCl_2_ and Selected D_2_ Dopaminergic Agonists on mRNA Expression Level of vegfaa, vegfr1 and vegfr2

**Figure 3:**
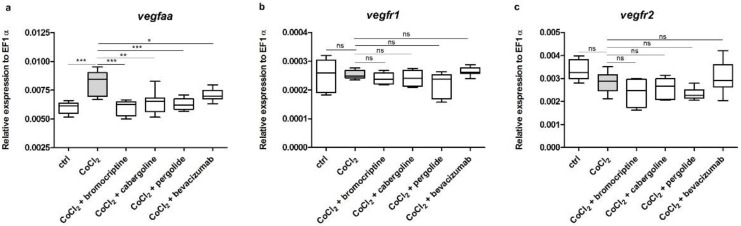
Expression profiles of zebrafish *vegfaa*, *vegfr1* and *vegfr2* genes. The graphs show data of the mRNA expression of (**a**) *vegfaa*, (**b**) *vegfr1* and (**c**) *vegfr2* from pooled 72-h post-fertilization (hpf) wild-type zebrafish larvae (*n* = 30) in six experimental groups: (1) control, (2) exposed to 5 mM CoCl_2_; (3) exposed to a mixture of 5 mM CoCl_2_ and 2.5 µM/L bromocriptine; (4) exposed to a mixture of 5 mM CoCl_2_ and 2.5 µM/L cabergoline; (5) exposed to a mixture of 5 mM CoCl_2_ and 2.5 µM/L pergolide; and (6) exposed to a mixture of 5 mM CoCl_2_ and 2.5 µM/L bevacizumab. Each group was covered by samples analyzed in triplicate in three separate experiments. Data in the figure represent the average of the three individual experiments. Gene expression values were normalized to housekeeping gene *ef1-α*; 5 mM CoCl_2_ exposure resulted in significant upregulation of the expression of *vegfaa* in comparison to the control group (**a**). Co-treatment with 2.5 µM/L bromocriptine, 2.5 µM/L cabergoline, 2.5 µM/L pergolide and 2.5 µM/L bevacizumab resulted in a statistically significant decrease in the expression of previously up regulated *vegfaa* (**a**). The expression of *vegfr1* and *vegfr2* were not altered by both 5 mM CoCl_2_ treatment and dopaminergic agonists’ co-treatment in comparison to the control (**b**). (One-way ANOVA, GraphPad Prism 5, *** *p* < 0.001; ** *p* < 0.01; * *p* < 0.05; ns: not statistically significant differences (*p* > 0.05)).

The exposure to 5 mM CoCl_2_ significantly upregulated the expression of *vegfaa* in comparison to that obtained in the control group (*p* < 0.001) (Figure 3a). Co-treatment with 2.5 µM/L bromocriptine, 2.5 µM/L cabergoline, 2.5 µM/L pergolide and 2.5 µM/L bevacizumab resulted in a statistically significant decrease in the expression of previously up-regulated *vegfaa*, thus equalizing it to the control value (*p* < 0.05–0.001) (Figure 3a). The 5 mM CoCl_2_ treatment as well as co-treatments with the investigated dopaminergic agonists did not significantly alter the expression of *vegfr1* and *vegfr2* in comparison to that obtained in the control group (*p* > 0.05) (Figure 3b,c).

### 3.4. Effects of CoCl_2_ and Selected D_2_ Dopaminergic Agonists on mRNA Expression Level of Chosen Growth Factors

**Figure 4:**
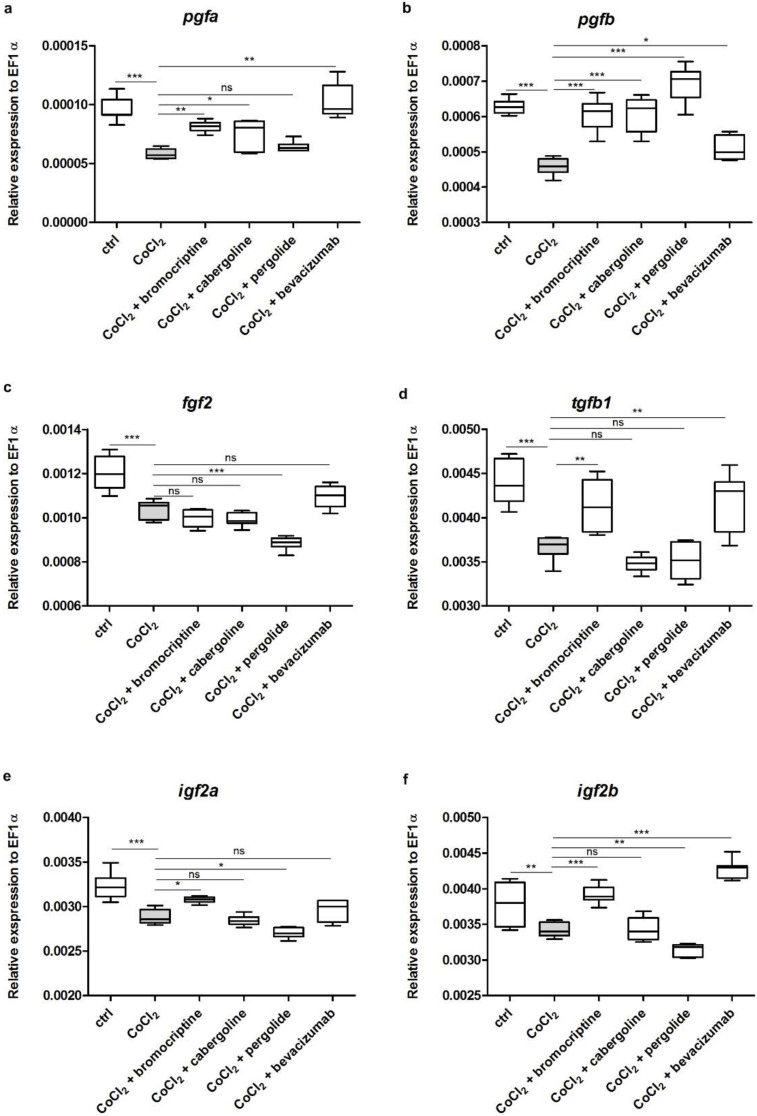
Expression profiles of zebrafish *pgfa*, *pgfb*, *fgf2*, *tgfb1*, *igf2a* and *igf2b* genes. The graphs show data of the mRNA expression of (**a**) *pgfa*, (**b**) *pgfb*, (**c**) *fgf2*, (**d**) *tgfb1a*, (**e**) *igf2a* and (**f**) *igf2b* from pooled 72-h post-fertilization (hpf) wild-type zebrafish larvae (*n* = 30) in six experimental groups: (1) control; (2) exposed to 5 mM CoCl_2_; (3) exposed to a mixture of 5 mM CoCl_2_ and 2.5 µM/L bromocriptine; (4) exposed to a mixture of 5 mM CoCl_2_ and 2.5 µM/L cabergoline; (5) exposed to a mixture of 5 mM CoCl_2_ and 2.5 µM/L pergolide; and (6) exposed to a mixture of 5 mM CoCl_2_ and 2.5 µM/L bevacizumab. Each group was covered by samples analyzed in triplicate in three separate experiments. Data in the figure represent the average of the three individual experiments. Gene expression values were normalized to housekeeping gene *ef1-α*; 5 mM CoCl_2_ exposure resulted in significant downregulation of the expression of all the above-mentioned genes in comparison to the control group (**a**–**f**). Co-treatment with 2.5 µM/L bromocriptine, 2.5 µM/L cabergoline, 2.5 µM/L pergolide and 2.5 µM/L bevacizumab resulted in a statistically significant increase of the expression of previously up downregulated *pgfa* and *pgfb* (**a**,**b**). Co-treatment with dopaminergic agonists has varied influence on genes encoding the chosen growth factors (see detailed description in the text) (**c**–**g**). (One-way ANOVA, GraphPad Prism 5, *** *p* < 0.001; ** *p* < 0.01; * *p* < 0.05; ns: not statistically significant differences (*p* > 0.05)).

The expression of all studied genes (*pgfa*, *pgfb*, *fgf2*, *tgfb1*, *igf2a*, *igf2b*) was statistically significantly downregulated by 5 mM CoCl_2_ treatment in comparison to the control group (*p* < 0.05–0.001) (Figure 4a–f). The CoCl_2_-derived lower expressions of *pgfa* and *pgfb* were significantly increased by co-treatment with 2.5 µM/L bromocriptine, 2.5 µM/L cabergoline, 2.5 µM/L pergolide and 2.5 µM/L bevacizumab, respectively (*p* < 0.05–0.001) (Figure 4a,b). *Fgf2* was not altered by dopaminergic agonists; only pergolide resulted in an even more severe decrease in comparison to the CoCl_2_ treated group (*p* < 0.001) (Figure 4c). The changes of the expression of *tgfb1*, *igfa* and *igf2b* were similar in all studied groups. Treatment with bromocriptine was the only one that resulted in increased expression (*p* < 0.05–0.001), while co-treatments with cabergoline and pergolide had neutral or reducing influence on mRNA levels of studied genes (*p* > 0.05, *p* < 0.05) (Figure 4d–f).

## 4. Discussion

Our study indicates that selected D_2_ dopaminergic receptor agonists, namely cabergoline, bromocriptine and pergolide, inhibit abnormal neovascularization of HRVs and ISVs in 3 dpf zebrafish larvae. Moreover, our results are comparable to those obtained with the use of bevacizumab—a well-studied anti-VEGF recombinant humanized monoclonal IgG antibody [15]. The qPCR analyses supported the protective role of the chosen dopaminergic receptor agonists by demonstrating their influence on CoCl_2_-derived up-regulation of VEGF gene expression.

We used CoCl_2_ to evoke hypoxia leading to rapid pathological neovascularization. This model of chemical hypoxia is quite well-known and used in zebrafish [16]. Cobalt ions (Co^+2^) replace iron ions (Fe^+2^) and stop the activity of propyl hydroxylases (PHDs) enzymes, which catalyze the breakdown of hypoxia inducible factors (HIFs) and directly bind HIFs to reduce their degradation [17,18]. Currently, three types of HIFs are known: 1alpha, 2alpha and 3alpha. However, only the HIF-1alpha biological effect is well described. HIF-1alpha modifies glucose and the Krebs pathways leading to the activation of many gene coding factors related to angiogenesis, erythropoiesis and cellular apoptosis, such as VEGF and erythropoietin [19,20]. Therefore, one of the possibilities is that HIF-1alpha released during chemical hypoxia increases VEGF production, which stimulates new blood vessels formation. Our results are consistent with previous studies which revealed that chemically-induced hypoxia resulted in VEGF gene overexpression followed by increased abnormal angiogenesis of zebrafish HRVs and ISVs and this effect was blocked by bevacizumab [21,22].

However, bevacizumab was not the main subject of our research and constituted a positive control. Our main investigations showed that selected D_2_ dopaminergic receptor agonists, bromocriptine, cabergoline and pergolide, block, stimulated by chemical hypoxia, *vegfaa* overexpression and have ameliorative influence on disrupted angiogenesis of HRVs and ISVs. The anti-angiogenic effect of dopamine and selected D_2_ agonists was also observed in vitro and in various animal models. It was showed that dopamine and D_2_ agonists inhibit, dependent on VEGF, new blood vessels formation and reduce vascular permeability within neoplastic tumors such as gastric cancer [23], colorectal cancer [24], malignant melanoma [25], bone marrow tumors [26], ovarian cancer [27], small cell carcinoma lung cancer [28], non-small cell lung cancer [29], prostate cancer [30] and a tumor of the pituitary gland [31]. Another study revealed that dopamine and dopaminergic D_2_ agonists decrease blood vessel permeability in animal model of ovarian hyperstimulation syndrome (OHSS), and this effect was related with the increase of VEGF secretion [32].

Interestingly, it was also presented that dopamine and dopaminergic D_1_ and D_2_ agonists agonists are unlikely to inhibit the proliferation of human umbilical vein endothelial cells (HUVEC), and this effect was related with the inhibition of VEGFR2 phosphorylation by increasing the level of Src-homology-2-domain-containing protein tyrosine phosphatase (SHP-2) [33,34,35].

We showed that selected D_2_ dopaminergic receptor agonists decrease *vegfaa* expression. The mechanism of how cabergoline, bromocriptine and pergolide decrease *vegfaa* expression remains unknown. It has been suggested by other authors that D_2_ agonists can reduce VEGF gene expression directly or indirectly through inhibition of HIF-1 alpha activity [36,37]. Moreover, there is still no evidence if the inhibitory effect of cabergoline, bromocriptine and pergolide on VEGF gene expression is related to activation of D_2_ receptors. It was only described previously that the above-mentioned D_2_ agonists have no effect on developmental angiogenesis of peripheral blood vessels in rat and human [38,39]. This was explained by the lack of the presence or activity of D_2_ dopaminergic receptors in the peripheral circulation at the fetal life. Our data do not give evidence for D_2_ dopaminergic receptor presence in HRVs and ISVs of zebrafish embryos or for the suppressive activity of cabergoline, bromocriptine and pergolide against VEGF independent of D_2_ receptor activation. According to Cacaveli [40], it is also possible that D_2_ receptors are variable and associated with many means of intracellular transmission, explaining various biological effects of their activity. VEGF itself can act through specific receptors. Currently, three types of VEGF receptors (VEGFRs) are known. VEGFR-1 and VEGFR-2 are found in vascular endothelial cells, but VEGFR-3 is mainly located in lymphatic endothelial cells and play a different role in the development and reactivity of blood vessels [41,42,43]. Our study shows that selected D_2_ dopaminergic agonists and bevacizumab have no effect on *vegfr1* and *vegfr2* expression in chemical hypoxia conditions. It is already well established that bevacizumab blocks VEGF-induced VEGFR-2 protein phosphorylation and has no effect on mRNA expression level [15]. Lack of effect of cabergoline, bromocriptine and pergolide on *vegfr2* expression suggest that the mode of action of selected D_2_ dopaminergic agonists is similar to bevacizumab. VEGFR-1 is considered as a non-signaling receptor but has an important biological role in regulating VEGF-mediated signaling through VEGFR-2. Therefore, it is highly possible that changes of VEGFR-1 expression are also detectable only on the protein level.

Even though VEGF is the key protein responsible for the development and maintenance of the vascular and lymphatic systems, there are many angiogenic and inflammatory agents which can stimulate angiogenesis regardless of VEGF. Among them we can mention fibroblast growth factors (FGFs), angiopoietin-1 and 2, transforming growth factor-beta 1 (TGF-beta 1), insulin-like growth factor 2 (IGF 2), placental growth factor (PGF), platelet-derived growth factor (PDGF), other interleukins and cytokines. Most of them have proven stimulative effects on retinal angiogenesis [44]. However, the question if D_2_ agonists can interact with those substances and modulate their angiogenetic properties remains open. To partially answer of this question, we investigated the mRNA expression levels of some above-mentioned biomarkers. We demonstrated that the expression of *pga* and *pgb* is strongly downregulated after CoCl_2_ treatment, and D_2_ dopaminergic agonists prevent this process. Despite PGF being a member of the VEGF family, its gene expression profile is opposite to that of VEGF. Our findings correspond to those obtained by Khaliq et al., who revealed that hypoxia promotes angiogenesis and upregulates VEGF expression while it downregulates PGF that possess 53% homology with VEGF [45]. Based on previous studies, we have chosen FGF2 as a major angiogenic factor involved in normal and pathological angiogenesis [46,47,48]. Our results showed its decreased mRNA expression after hypoxic conditions. A study by Conte et al. confirms these observations and demonstrates interesting discrepancy between FGF_2_ mRNA and protein level [49]. They claim that FGF2 is induced at the protein level during ischemia, concomitant with HIF-1α induction and a decrease in FGF2 mRNA. However, the opposite effects are also already described [50]. In our study, only pergolide significantly altered *fgf2* level, decreasing it even more in comparison to the CoCl_2_-exposed group. Hypoxia is thought to promote TGF-beta 1 and IGF2 posttranslational expression and secretion [51,52], but mRNA susceptibility is moderate [53,54,55]. Our results show that both *tgfb1* and *igf2a*/*igf2b* genes are downregulated in CoCl_2_ treated groups. The divergences may result from the fact that our study was performed on embryos and concerned chronic hypoxia conditions. This interesting finding provides new insight into *tgfb1* and *igf2a*/*igf2b* regulation under hypoxia conditions. Additionally, our findings concerning IGF2 mRNA expression fully correspond with those obtained by Eme et al. [55], who studied D_2_ dopaminergic agonists of the above-mentioned differently regulated markers. Bromocriptine had a strong increasing impact, whereas cabergoline and pergolide had a decreasing or no impact on previously downregulated genes. The varied expression values determined in individual experimental groups may be due to the fact that selected D_2_ agonists have different receptor affinities. Bromocriptine is a strong D_2_ agonist, while cabergoline and pergolide have weaker D_2_ affinity, with simultaneous D_1_ affinity [56]. Despite the fact that the results obtained on mRNA expression level shed new light on potential properties of D2 agonists, the qPCR method is not sufficient to describe the complex molecular mechanism of d2 agonists’ action. However, due to the lack of zebrafish specific antibodies, investigations on the protein level are also hampered, and this obstacle needs to be overcome in the future.

Pathological retinal angiogenesis plays a crucial role in etiopathogenesis of various VEGF-dependent retinal diseases, such as age-related macular degeneration (AMD), proliferative diabetic retinopathy (PDR), diabetic macular edema (DMO), central retinal vein occlusion (CRVO) or retinopathy of prematurity (ROP), which are the main causes of serious vision impartment or blindness in human. Nowadays there are only a few anti-VEGF drugs available for their treatment; therefore, further investigations need to be conducted to discover more effective, comfortable in use and cheaper anti-VEGF drugs. Zebrafish embryos and larvae seem to be a very good model for the identification of novel inhibitors of retinal pathological angiogenesis. Even though zebrafish are not able to spontaneously develop vascular diseases analogous to those seen in humans, similarity of genes and mechanisms of blood vessel formation to other vertebrates, the simple method of adding drugs, good techniques of eye vasculature visualization and availability of fluorescent transgenic lines of zebrafish facilitate initial testing of antiangiogenic substances [22,57]. However, it is challenging to evoke all syndromes of disease in zebrafish. This model is instead used to study isolated mechanism thought to play a role in human whole spectrum disease. In terms of our study, it seems that hypoxia is one amongst various factors including oxidative stress, inflammation and disfunction of endothelial cells, which can lead to pathological angiogenesis in VEGF-dependent retinal diseases. Therefore, the observed antiangiogenic effect of selected substances should be confirmed in other experimental in vitro and in vivo models.

## 5. Conclusions

In conclusion, from our study emerged three D_2_ dopaminergic receptor agonists, namely bromocriptine, cabergoline and pergolide, which should be taken under consideration as a potential group of new anti-VEGF drugs used in ophthalmology. We demonstrated that D2 dopaminergic agonists are able to smooth out morphological CoCl_2_-derived defects in newly formed HRVs and ISVs as well as downregulate *vegfaa* overexpression. The obtained results unequivocally prove that bromocriptine, cabergoline and pergolide are important factors playing a role in VEGF-dependent blood vessel formation, and this interesting property is not limited only to the eye vasculature. Therefore, it cannot be excluded that the studied D_2_ agonists have more universal properties and could be used in the treatment of other vasculature diseases.

## Figures and Tables

**Table 1:** Primers used in the study.

Gene	Forward 5′ -3′	Reverse 5′-3′	Accession No.
*ef-1*α	CTGGAGGCCAGCTCAAACAT	ATCAAGAAGAGTAGTACCGCTAGCATTAC	NM_131263.1
*vegfaa*	CTGCTGGTAGACATCATC	TTTCGTGTCTCTGTCGGG	XM_009292018.3, NM_131408.3, NM_001110349.2
*vegfr1*	AGCCACAGACAGGAAGTGTT	GACACAGCGATAGATGCCAGA	NM_001014829.3
*vegfr2*	CTGCTGGTAGACATCATC	TTTCGTGTCTCTGTCGGG	XM_009291142.3
*pgfa*	GCTGCTGCAACGACGAAAAA	CATAATCTCTGCGCCGCTCT	XM_021468740.1
*pgfb*	ACCTACAACAAAACAAGACAGATGG	GAGACAGCGTTTACCTGCGG	XM_017351845.2
*fgf2*	GCATCTGTACCAACCGTTTCC	ATCTGTGGTCCTTTTCGTCCC	NM_212823.2
*tgfb1*	TGTACCCGCAATCCTTGACC	GGACAATTGCTCCACCTTGTG	NM_182873.1
*igf2a*	ACAGGCTCTTCACAAGGACAC	TTCGGGCCAACAGAATGGAT	NM_131433.1
*igf2b*	CGCATTAAAACAGGAGGTCCC	CTGAGCAGCCTTTCTTTGCC	NM_001001815.1

## Data Availability

Data available upon request from the authors.

## References

[1] Maniscalco P.W.M., D’Angio C.T. (2006). Vascular endothelial growth factor. Encyclopedia of Respiratory Medicine.

[2] Apte R.S., Chen D.S., Ferrara N. (2019). VEGF in Signaling and Disease: Beyond Discovery and Development. Cell.

[3] Campochiaro P.A. (2015). Molecular pathogenesis of retinal and choroidal vascular diseases. Prog. Retin. Eye Res..

[4] Hartnett M.E. (2020). Retinopathy of Prematurity: Evolving Treatment With Anti-Vascular Endothelial Growth Factor. Am. J. Ophthalmol..

[5] Chen P.J., Wan L., Lai J.N., Chen C.S., Chen J.J., Yen W.M., Chiu L.T., Hu K.C., Tien P.T., Lin H.J. (2021). Increased risk of Parkinson’s disease among patients with age-related macular degeneration. BMC Ophthalmol..

[6] Brilliant M.H., Vaziri K., Connor T.B., Schwartz S.G., Carroll J.J., McCarty C.A., Schrodi S.J., Hebbring S.J., Kishor K.S., Flynn H.W. (2016). Mining Retrospective Data for Virtual Prospective Drug Repurposing: L-DOPA and Age-related Macular Degeneration. Am. J. Med..

[7] Tang H., Mourad S.M., Wang A., Zhai S.D., Hart R.J. (2021). Dopamine agonists for preventing ovarian hyperstimulation syndrome. Cochrane Database Syst. Rev..

[8] Herbert R.C., Thompson D.L., Mitcham P.B., Lestelle J.D., Gilley R.M., Burns P.J. (2013). Inhibitory Effects of Pergolide and Cabergoline Formulations on Daily Plasma Prolactin Concentrations in Geldings and on the Daily Prolactin Responses to a Small Dose of Sulpiride in Mares. J. Equine Vet. Sci..

[9] Bonuccelli U., Colzi A., Del Dotto P. (2002). Pergolide in the treatment of patients with early and advanced Parkinson’s disease. Clin. Neuropharmacol..

[10] Chávez M.N., Aedo G., Fierro F.A., Allende M.L., Egaña J.T. (2016). Zebrafish as an Emerging Model Organism to Study Angiogenesis in Development and Regeneration. Front. Physiol..

[11] Xiong J.W. (2008). Molecular and developmental biology of the hemangioblast. Dev. Dyn..

[12] Eberlein J., Herdt L., Malchow J., Rittershaus A., Baumeister S., Helker C.S. (2021). Molecular and Cellular Mechanisms of Vascular Development in Zebrafish. Life.

[13] Kimmel C.B., Ballard W.W., Kimmel S.R., Ullmann B., Schilling T.F. (1995). Stages of embryonic development of the zebrafish. Dev. Dyn..

[14] Kasica N., Jakubowski P., Kaleczyc J. (2021). P-Glycoprotein Inhibitor Tariquidar Plays an Important Regulatory Role in Pigmentation in Larval Zebrafish. Cells.

[15] Zhang J., Gao B., Zhang W., Qian Z., Xiang Y. (2018). Monitoring antiangiogenesis of bevacizumab in zebrafish. Drug Des. Dev. Ther..

[16] Wu Y.C., Chang C.Y., Kao A., Hsi B., Lee S.H., Chen Y.H., Wang I.J. (2015). Hypoxia-induced retinal neovascularization in zebrafish embryos: A potential model of retinopathy of prematurity. PLoS ONE.

[17] Epstein A.C., Gleadle J.M., McNeill L.A., Hewitson K.S., O’Rourke J., Mole D.R., Mukherji M., Metzen E., Wilson M.I., Dhanda A. (2001). *C. elegans* EGL-9 and mammalian homologs define a family of dioxygenases that regulate HIF by prolyl hydroxylation. Cell.

[18] Yuan Y., Hilliard G., Ferguson T., Millhorn D.E. (2003). Cobalt inhibits the interaction between hypoxia-inducible factor-alpha and von Hippel-Lindau protein by direct binding to hypoxia-inducible factor-alpha. J. Biol. Chem..

[19] Muñoz-Sánchez J., Chánez-Cárdenas M.E. (2019). The use of cobalt chloride as a chemical hypoxia model. J. Appl. Toxicol..

[20] Wu J., Ke X., Wang W., Zhang H., Ma N., Fu W., Zhao M., Gao X., Hao X., Zhang Z. (2016). Aloe-emodin suppresses hypoxia-induced retinal angiogenesis via inhibition of HIF-1α/VEGF pathway. Int. J. Biol. Sci..

[21] Alvarez Y., Astudillo O., Jensen L., Reynolds A.L., Waghorne N., Brazil D.P., Cao Y., O’Connor J.J., Kennedy B.N. (2009). Selective inhibition of retinal angiogenesis by targeting PI3 kinase. PLoS ONE.

[22] Chimote G., Sreenivasan J., Pawar N., Subramanian J., Sivaramakrishnan H., Sharma S. (2014). Comparison of effects of anti-angiogenic agents in the zebrafish efficacy-toxicity model for translational anti-angiogenic drug discovery. Drug Des. Dev. Ther..

[23] Chakroborty D., Sarkar C., Mitra R.B., Banerjee S., Dasgupta P.S., Basu S. (2004). Depleted dopamine in gastric cancer tissues: Dopamine treatment retards growth of gastric cancer by inhibiting angiogenesis. Clin. Cancer Res..

[24] Basu S., Dasgupta P.S. (1999). Decreased dopamine receptor expression and its second-messenger cAMP in malignant human colon tissue. Dig. Dis. Sci..

[25] Wick M.M., Kramer R.A., Gorman M. (1978). Enhancement of L-dopa incorporation into melanoma by dopa decarboxylase inhibition. J. Investig. Dermatol..

[26] Chakroborty D., Chowdhury U.R., Sarkar C., Baral R., Dasgupta P.S., Basu S. (2008). Dopamine regulates endothelial progenitor cell mobilization from mouse bone marrow in tumor vascularization. J. Clin. Investig..

[27] Moreno-Smith M., Lu C., Shahzad M.M., Pena G.N., Allen J.K., Stone R.L., Mangala L.S., Han H.D., Kim H.S., Farley D. (2011). Dopamine blocks stress-mediated ovarian carcinoma growth. Clin. Cancer Res..

[28] Senogles S.E. (2007). D2 dopamine receptor-mediated antiproliferation in a small cell lung cancer cell line, NCI-H69. Anti-Cancer Drugs.

[29] Roy S., Lu K., Nayak M.K., Bhuniya A., Ghosh T., Kundu S., Ghosh S., Baral R., Dasgupta P.S., Basu S. (2017). Activation of D2 Dopamine Receptors in CD133+ve Cancer Stem Cells in Non-small Cell Lung Carcinoma Inhibits Proliferation, Clonogenic Ability, and Invasiveness of These Cells. J. Biol. Chem..

[30] Chakroborty D., Sarkar C., Yu H., Wang J., Liu Z., Dasgupta P.S., Basu S. (2011). Dopamine stabilizes tumor blood vessels by up-regulating angiopoietin 1 expression in pericytes and Kruppel-like factor-2 expression in tumor endothelial cells. Proc. Natl. Acad. Sci. USA.

[31] Chauvet N., Romanò N., Lafont C., Guillou A., Galibert E., Bonnefont X., Le Tissier P., Fedele M., Fusco A., Mollard P. (2017). Complementary actions of dopamine D2 receptor agonist and anti-vegf therapy on tumoral vessel normalization in a transgenic mouse model. Int. J. Cancer.

[32] Ferrero H., García-Pascual C.M., Gómez R., Delgado-Rosas F., Cauli O., Simón C., Gaytán F., Pellicer A. (2014). Dopamine receptor 2 activation inhibits ovarian vascular endothelial growth factor secretion in vitro: Implications for treatment of ovarian hyperstimulation syndrome with dopamine receptor 2 agonists. Fertil. Steril..

[33] Basu S., Nagy J.A., Pal S., Vasile E., Eckelhoefer I.A., Bliss V.S., Manseau E.J., Dasgupta P.S., Dvorak H.F., Mukhopadhyay D. (2001). The neurotransmitter dopamine inhibits angiogenesis induced by vascular permeability factor/vascular endothelial growth factor. Nat. Med..

[34] Sarkar C., Chakroborty D., Mitra R.B., Banerjee S., Dasgupta P.S., Basu S. (2004). Dopamine in vivo inhibits VEGF-induced phosphorylation of VEGFR-2, MAPK, and focal adhesion kinase in endothelial cells. Am. J. Physiol. Heart Circ. Physiol..

[35] Sinha S., Vohra P.K., Bhattacharya R., Dutta S., Sinha S., Mukhopadhyay D. (2009). Dopamine regulates phosphorylation of VEGF receptor 2 by engaging Src-homology-2-domain-containing protein tyrosine phosphatase 2. J. Cell Sci..

[36] Büchler P., Reber H.A., Büchler M.W., Friess H., Lavey R.S., Hines O.J. (2004). Antiangiogenic activity of genistein in pancreatic carcinoma cells is mediated by the inhibition of hypoxia-inducible factor-1 and the down-regulation of VEGF gene expression. Cancer.

[37] Beckert S., Farrahi F., Perveen Ghani Q., Aslam R., Scheuenstuhl H., Coerper S., Königsrainer A., Hunt T.K., Hussain M.Z. (2006). IGF-I-induced VEGF expression in HUVEC involves phosphorylation and inhibition of poly(ADP-ribose)polymerase. Biochem. Biophys. Res. Commun..

[38] Narburgh L.J., Turner J., Freeman S.J. (1990). Evaluation of the teratogenic potential of the dopamine agonist bromocriptine in rats. Toxicol. Lett..

[39] Turkalj I., Braun P., Krupp P. (1982). Surveillance of bromocriptine in pregnancy. JAMA.

[40] Caccavelli L., Cussac D., Pellegrini I., Audinot V., Jaquet P., Enjalbert A. (1992). D2 dopaminergic receptors: Normal and abnormal transduction mechanisms. Horm. Res..

[41] Stuttfeld E., Ballmer-Hofer K. (2009). Structure and function of VEGF receptors. IUBMB Life.

[42] Matsumoto K., Ema M. (2014). Roles of VEGF-A signalling in development, regeneration, and tumours. J. Biochem..

[43] Ober E.A., Olofsson B., Mäkinen T., Jin S.W., Shoji W., Koh G.Y., Alitalo K., Stainier D.Y. (2004). Vegfc is required for vascular development and endoderm morphogenesis in zebrafish. EMBO Rep..

[44] Rezzola S., Belleri M., Gariano G., Ribatti D., Costagliola C., Semeraro F., Presta M. (2014). In vitro and ex vivo retina angiogenesis assays. Angiogenesis.

[45] Khaliq A., Dunk C., Jiang J., Shams M., Li X.F., Acevedo C., Weich H., Whittle M., Ahmed A. (1999). Hypoxia down-regulates placenta growth factor, whereas fetal growth restriction up-regulates placenta growth factor expression: Molecular evidence for “placental hyperoxia” in intrauterine growth restriction. Lab. Investig..

[46] Poole T.J., Finkelstein E.B., Cox C.M. (2001). The role of FGF and VEGF in angioblast induction and migration during vascular development. Dev. Dyn..

[47] Jia T., Jacquet T., Dalonneau F., Coudert P., Vaganay E., Exbrayat-Héritier C., Vollaire J., Josserand V., Ruggiero F., Coll J.L. (2021). FGF-2 promotes angiogenesis through a SRSF1/SRSF3/SRPK1-dependent axis that controls VEGFR1 splicing in endothelial cells. BMC Biol..

[48] Javerzat S., Auguste P., Bikfalvi A. (2002). The role of fibroblast growth factors in vascular development. Trends Mol. Med..

[49] Conte C., Riant E., Toutain C., Pujol F., Arnal J.F., Lenfant F., Prats A.C. (2008). FGF2 translationally induced by hypoxia is involved in negative and positive feedback loops with HIF-1alpha. PLoS ONE.

[50] Kakudo N., Morimoto N., Ogawa T., Taketani S., Kusumoto K. (2015). Hypoxia Enhances Proliferation of Human Adipose-Derived Stem Cells via HIF-1ɑ Activation. PLoS ONE.

[51] Mingyuan X., Qianqian P., Shengquan X., Chenyi Y., Rui L., Yichen S., Jinghong X. (2017). Hypoxia-inducible factor-1α activates transforming growth factor-β1/Smad signaling and increases collagen deposition in dermal fibroblasts. Oncotarget.

[52] Marques I.J., Leito J.T., Spaink H.P., Testerink J., Jaspers R.T., Witte F., van den Berg S., Bagowski C.P. (2008). Transcriptome analysis of the response to chronic constant hypoxia in zebrafish hearts. J. Comp. Physiol. B.

[53] Scheid A., Wenger R.H., Christina H., Camenisch I., Ferenc A., Stauffer U.G., Gassmann M., Meuli M. (2000). Hypoxia-regulated gene expression in fetal wound regeneration and adult wound repair. Pediatr. Surg. Int..

[54] Khaidakov M., Szwedo J., Mitra S., Mehta J.L. (2011). Angiostatic effects of aspirin in hypoxia-reoxygenation are linked to modulation of TGFβ1 signaling. J. Cardiovasc. Pharmacol. Ther..

[55] Eme J., Rhen T., Tate K.B., Gruchalla K., Kohl Z.F., Slay C.E., Crossley D.A. (2013). Plasticity of cardiovascular function in snapping turtle embryos (Chelydra serpentina): Chronic hypoxia alters autonomic regulation and gene expression. Am. J. Physiol. Regul. Integr. Comp. Physiol..

[56] Ohta K., Kuno S., Mizuta I., Fujinami A., Matsui H., Ohta M. (2003). Effects of dopamine agonists bromocriptine, pergolide, cabergoline, and SKF-38393 on GDNF, NGF, and BDNF synthesis in cultured mouse astrocytes. Life Sci..

[57] Rezzola S., Paganini G., Semeraro F., Presta M., Tobia C. (2016). Zebrafish (Danio rerio) embryo as a platform for the identification of novel angiogenesis inhibitors of retinal vascular diseases. Biochim. Biophys. Acta.

